# TGFβ-mediated suppression of CD248 in non-cancer cells via canonical Smad-dependent signaling pathways is uncoupled in cancer cells

**DOI:** 10.1186/1471-2407-14-113

**Published:** 2014-02-20

**Authors:** Sahana Suresh Babu, Yanet Valdez, Andrea Xu, Alice M O’Byrne, Fernando Calvo, Victor Lei, Edward M Conway

**Affiliations:** 1 Centre for Blood Research, Department of Medicine, University of British Columbia, 4306-2350 Health Sciences Mall, V6T 1Z3, BC Vancouver, Canada; 2 Tumour Cell Biology Laboratory, Cancer Research UK London Research Institute, London, UK; 3 Tumour Microenvironment Team Division of Cancer Biology, The Institute of Cancer Research, London, UK

## Abstract

**Background:**

CD248 is a cell surface glycoprotein, highly expressed by stromal cells and fibroblasts of tumors and inflammatory lesions, but virtually undetectable in healthy adult tissues. CD248 promotes tumorigenesis, while lack of CD248 in mice confers resistance to tumor growth. Mechanisms by which CD248 is downregulated are poorly understood, hindering the development of anti-cancer therapies.

**Methods:**

We sought to characterize the molecular mechanisms by which CD248 is downregulated by surveying its expression in different cells in response to cytokines and growth factors.

**Results:**

Only transforming growth factor (TGFβ) suppressed CD248 protein and mRNA levels in cultured fibroblasts and vascular smooth muscle cells in a concentration- and time-dependent manner. TGFβ transcriptionally downregulated CD248 by signaling through canonical Smad2/3-dependent pathways, but not via mitogen activated protein kinases p38 or ERK1/2. Notably, cancer associated fibroblasts (CAF) and cancer cells were resistant to TGFβ mediated suppression of CD248.

**Conclusions:**

The findings indicate that decoupling of CD248 regulation by TGFβ may contribute to its tumor-promoting properties, and underline the importance of exploring the TGFβ-CD248 signaling pathway as a potential therapeutic target for early prevention of cancer and proliferative disorders.

## Background

CD248, also referred to as endosialin and tumor endothelial marker (TEM-1) [1] (reviewed in [2]), is a member of a family of type I transmembrane glycoproteins containing C-type lectin-like domains, that includes thrombomodulin [3] and CD93 [4]. Although the mechanisms are not fully elucidated, these molecules all modulate innate immunity, cell proliferation and vascular homeostasis and are potential therapeutic targets for several diseases, including cancer, inflammatory disorders and thrombosis.

CD248 is expressed by cells of mesenchymal origin, including murine embryonic fibroblasts (MEF), vascular smooth muscle cells, pericytes, myofibroblasts, stromal cells and osteoblasts [5-12]. During embryonic development, CD248 is prominently and widely expressed in the fetus (reviewed in [2]). However, after birth, CD248 protein levels are dramatically downregulated [7,13-15], resulting in only minimal expression in the healthy adult, except in the endometrium, ovary, renal glomerulus and osteoblasts [11,16-18].

While largely absent in normal tissues, CD248 is markedly upregulated in almost all cancers. Highest expression is found in neuroblastomas and in subsets of carcinomas, such as breast and colon cancers, and in addition, in glioblastomas and mesenchymal tumors, such as fibrosarcomas and synovial sarcomas [8,14,15,17,19,20], where it is mostly detected in perivascular and tumor stromal cells, but also in the tumor cells themselves [21,22]. CD248 is also expressed in placenta and during wound healing and in wounds such as ulcers. It is also prominently expressed in synovial fibroblasts during inflammatory arthritis [10]. In some tumors and in chronic kidney disease, CD248 expression directly correlates with worse disease and/or a poor prognosis [9,23,24]. The contributory role of CD248 to these pathologies was confirmed in gene inactivation studies. Mice lacking CD248 are generally healthy, except for an increase in bone mass [11,25] and incomplete post-natal thymus development [26]. However, in several models, they are protected against tumor growth, tumor invasiveness and metastasis [25,27] and they are less sensitive to anti-collagen antibody induced arthritis [10].

While the mechanisms by which CD248 promotes tumorigenesis and inflammation are not clearly defined, the preceding observations have stimulated interest in exploring CD248 as a therapeutic target, primarily by using anti-CD248 antibodies directed against its ectodomain [19,20,28,29]. Likely due to limited knowledge of CD248 regulatory pathways, other approaches to interfere with or suppress CD248 have not been reported. CD248 is upregulated *in vitro* by high cell density, serum starvation, by the oncogene *v-mos*[5] and by hypoxia [30]. We previously showed that fibroblast expression of CD248 is suppressed by contact with endothelial cells [27]. Otherwise, factors which down-regulate CD248 have not heretofore been reported, yet such insights might reveal novel sites for therapeutic intervention.

In this study, we evaluated the effects of several cytokines on the expression of CD248. We show that TGFβ specifically and dramatically downregulates CD248 expression in normal cells of mesenchymal origin and that this is mediated via canonical Smad-dependent intracellular signaling pathways. Notably, cancer cells and cancer associated fibroblasts are resistant to TGFβ mediated suppression of CD248. The findings suggest that CD248 not only promotes tumorigenesis, but may be a marker of the transition of TGFβ from a tumor suppressor to a tumor promoter. Delineating the pathways that couple TGFβ and CD248 may uncover novel therapeutic strategies.

## Methods

### Reagents

Rabbit anti-human CD248 antibodies (Cat no #18160-1AP) were from ProteinTech (Chicago, USA); goat anti-human actin antibodies (#sc-1616) from Santa Cruz (USA); rabbit anti-SMAD1,5-Phospho (Cat no #9516), rabbit anti-Smad2-Phospho (#3101), rabbit anti-ERK1/2-phospho (#9101S), rabbit anti-p38-phospho (#9211), rabbit anti-SMAD2/3 (#5678) and rabbit anti-SMAD3 (#9513) were from Cell Signaling (USA). Murine anti-rabbit α-smooth muscle actin monoclonal antibodies (#A5228) were from Sigma-Aldrich (Canada). Secondary antibodies included goat anti-rabbit IRDye® 800 (LIC-926-32211). Goat anti-rabbit IRDye® 680 (LIC-926-68071) or donkey anti-goat IRDye® 680 antibodies (LIC-926-68024) and anti-rabbit Alexa green-488 were from Licor (Nebraska, USA).

Basic fibroblast growth factor (bFGF), recombinant human transforming growth factor β-1 (TGFβ) (240-B/CF), recombinant human bone morphogenic protein (BMP-2) (355-BM-010/CF), recombinant human/mouse/Rat Activin A, CF (338-AC-010/CF), recombinant rat platelet derived growth factor-BB (PDGF) (250-BB-050), recombinant human vascular endothelial growth factor (VEGF), and recombinant mouse interleukin-6 (IL-6) (406-ML/CF), recombinant mouse tumor necrosis factor-α (TNF-α) (410-MT/CF) and recombinant mouse interferon-γ (IFN-γ) (485-MI/CF) were purchased from R&D Systems (Minneapolis, USA). Phorbol 12-Myristate 13-Acetate (PMA) (P1585) and α-amanitin were from Sigma-Aldrich (Oakville, Canada). The inhibitors SB431542 (for ALK5), SB202190 (for p38) and U0126 (for ERK1/2) were from Tocris Biosciences, Canada.

### Mice

Transgenic mice lacking CD248 (CD248^KO/KO^) were previously generated and genotyped as described [10]. Mice were maintained on a C57Bl6 genetic background and corresponding sibling-derived wild-type mice (CD248^WT/WT^) were used as controls.

### Cell culture

Murine embryonic fibroblasts (MEF) were isolated from CD248^WT/WT^ or CD248^KO/KO^ mice as previously described [10]. Cells were cultured in DMEM (Invitrogen, Canada) with 10% fetal calf serum (FCS) and 1% Penicillin/Streptomycin (Invitrogen, Karlsruhe, Germany) and used at passages 2-5. Upon reaching confluence, cells were incubated for 14 hrs in low serum media (1% FCS) and then treated as indicated in the Results with TGFβ (0.1-12 ng/ml), BMP-2 (50-100 ng/ml), PDGF (50 ng/ml), VEGF (20 ng/ml), bFGF (10 ng/ml), IL-6 10 ng/ml), PMA (60 ng/ml), SB43152 (1 μM), and/or α-amanitin (20 μg/ml), for different time periods as noted. Using previously reported methods [31,32], vascular smooth muscle cells (SMC) were isolated from the aortae of CD248^WT/WT^ or CD248^KO/KO^ pups, cultured in SMC growth media (Promocell, Heidelberg, Germany) with 15% FCS and 1% Penicillin/Streptomycin (Invitrogen) and used at passages 2-5. Wehi-231 and A20 (mouse B-lymphoma) cell lines (gift of Dr. Linda Matsuuchi, University of British Columbia) were cultured in RPMI media with 10% fetal calf serum (FCS), 1% Penicillin/Streptomycin and 0.1% mercaptoethanol. Normal fibroblasts (NF) derived from normal mouse mammary glands, and cancer associated fibroblasts (CAF) from mammary carcinoma in mice containing the MMTV-PyMT transgene [33] were provided by Dr. Erik Saha (Cancer Research London UK Research Institute, London, UK), and cultured in DMEM with 10% FCS, 1% Penicillin/Streptomycin and 1% insulin-transferrin-selenium.

### Protein electrophoresis and western blotting

Cells were scraped from culture dishes, suspended in PBS, pelleted by centrifugation and lysed with 50 μl RIPA buffer (30 mM Tris–HCl, 15 mM NaCl, 1% Igepal, 0.5% deoxycholate, 2 mM EDTA, 0.1% SDS). Centrifugation-cleared lysates were quantified for protein content. Equal quantities of cell lysates (25 μg) were separated by SDS-PAGE under reducing or non-reducing conditions as noted, using 8% and 12% low-bisacrylamide gels (acrylamide to bis-acrylamide = 118:1). In pilot studies, these gels provided highest resolution of the bands of interest [34]. Proteins were transferred to a nitrocellulose membrane and after incubating with blocking buffer (1:1 PBS:Odyssey buffer) (Licor, Nebraska, U.S.A.), they were probed with rabbit anti-CD248 antibodies 140 μg/ml, goat anti-actin antibodies, rabbit anti-Smad1-Phospho, anti-Smad2-Phospho, anti-Smad2-Total or anti-Smad3 antibodies in blocking buffer overnight. After washing and incubation of the filter with the appropriate secondary antibodies (100 ng/ml IRDye® 800 goat anti-rabbit or IRDye® Donkey anti-goat–Licor, Nebraska, USA) in blocking buffer for 1 hr at room temperature, detection was accomplished using a Licor Odyssey® imaging system (Licor, Nebraska, USA) and intensity of bands of interest were quantified relative to actin using Licor software (Licor, Nebraska, U.S). All studies were performed a minimum of 3 times, and representative Western blots are shown.

### Immunofluorescence analysis

Preconfluent cells were grown on cover slips and fixed at room temperature with acetone (100%) for 2 minutes, followed by a 30 minute incubation with blocking buffer (1% BSA in PBS). Cells were then incubated with anti-CD248 rabbit antibodies 40 μg/ml, for 1 hr followed by extensive washes and incubation with Alexa green 488 anti-rabbit antibody (5 mg/ml) for 1 hr. The cells were washed and fixed with antifade containing DAPI (Invitrogen, Canada) for subsequent imaging with a confocal microscopic (Nikon C2 model, Nikon, Canada).

### Determination of stability of CD248 mRNA

α-Amanitin, an inhibitor of RNA-polymerase II, was used to quantify the half-life of CD248 mRNA using previously reported methods [35]. Briefly, 90% confluent MEF were incubated with DMEM with 1% fetal calf serum (FCS) overnight, after which the media was refreshed, and subsequently stimulated with α-Amanitin 20 μg/ml ± TGFβ for the indicated time periods. RNA was isolated for gene expression analysis.

### Gene expression analysis

RNA was isolated from the MEF and reverse transcribed to cDNA/mRNA according to the manufacturer’s instructions (Qiagen RNeasy kit and QuantiTech reverse transcription kit, Hilden, Germany). Expression of CD248 mRNA was analyzed by RT-PCR and quantified with SYBR green using real time PCR (Applied Biosystems® Real-Time PCR Instrument, Canada). CD248 mRNA levels were reported relative to the expression of the housekeeping gene, Glyceraldehyde 3-Phosphate dehydrogenase (GAPDH). The following amplification primers were used: CD248 forward (5′-GGGCCCCTACCACTCCTCAGT-3′); CD248 reverse (5′-AGGTGGGTGGACAGGGCTCAG-3′); GAPDH forward (5′-GACCACAGTCCATGCCATCACTGC-3′); GAPDH reverse (5′-ATGACCTTGCCCACAGCCTTGG-3′).

### Animal care

Experimental animal procedures were approved by the Institutional Animal Care Committee of the University of British Columbia.

### Statistics

Experiments were performed in triplicate and data were analyzed using Bonferroni post-test to compare replicates (GraphPad Prism software Inc, California, USA). Error bars on figures represent standard errors of the mean (SEM). P < 0.05 was considered statistically significant.

## Results

### Screen for cytokines that modulate expression of CD248

**Figure 1:**
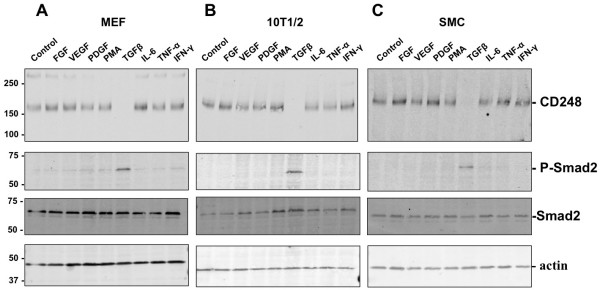
**Expression of CD248 by mesenchymal cells in response to cytokines and growth factors.** Murine embryonic fibroblasts (MEF) **(A)**, 10 T1/2 cells **(B)** and murine aortic smooth muscle cells (SMC) **(C)** were incubated for 48 hrs with FGF (10 ng/ml), VEGF (20 ng/ml), PDGF (20 ng/ml), PMA (60 ng/ml), TGFβ (3 ng/ml), IL-6 (10 ng/ml), TNF-α (10 ng/ml), or IFN-γ (10 ng/ml). Cells were lysed and separated by SDS-PAGE under non-reducing conditions for Western immunoblotting to detect CD248 and phosphorylated Smad2. Equal loading was confirmed with actin control. Only TGFβ suppressed expression of CD248, while inducing phosphorylation of Smad2. Results are representative of 3 independent experiments. Molecular weight markers in kDa are shown on the left.

In view of the established links between CD248 and cell proliferation, migration and invasion, we screened a number of growth factors, cytokines and PMA for effects on the expression of CD248 by MEF. These factors and the chosen concentrations were selected based on the fact that all reportedly induce MEF to undergo inflammatory, migratory and/or proliferative changes. We previously determined that these cells express CD248 at readily detectable levels, as assessed by Western blot, where it is often seen as a monomer (~150 kDa) and a dimer (~300 kDa). An incubation time of 48 hrs was chosen based on our previous findings that CD248-dependent release and activation of matrix metalloproteinase (MMP9) induced by TFGβ was observed over that period [10]. As seen in Figure 1A, bFGF, VEGF, PDGF, PMA, IL-6, TNF-α, and IFN-γ had no effects on CD248 expression. However, TGFβ suppressed expression of CD248 in MEF to almost undetectable levels (Figure 1A). The same pattern of response was evident in the murine fibroblast cell line 10 T1/2 (Figure 1B), and in mouse primary aortic smooth muscle cells (SMC) (Figure 1C), suggesting that CD248 specifically responds to TGFβ and that the response is active in diverse cell lines.

### TGFβ suppresses expression of CD248 by MEF

**Figure 2:**
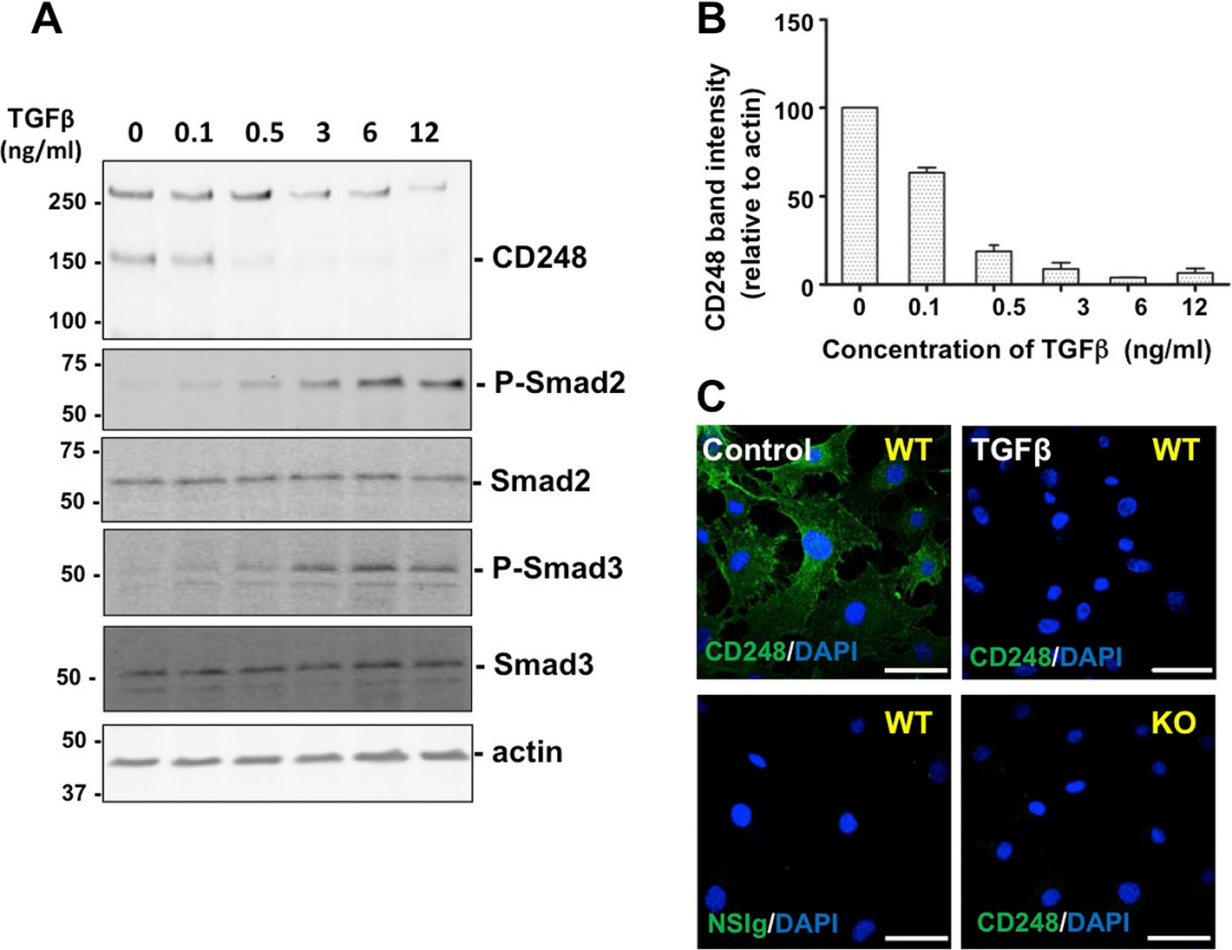
**Expression of CD248 in response to increasing concentrations of TGFβ. (A)** MEF were incubated for 48 hrs with increasing concentrations of TGFβ. Expression of CD248 (seen as monomers (~160 kDa) and dimers) and phosphorylation of Smad2, were detected by Western blot. **(B)** CD248 expression relative to actin expression was quantified by densitometry (n = 3 experiments) and results were normalized to the no-treatment condition. **(C)** CD248 expression by MEF (wild-type, WT; or lacking CD248, KO) was detected with specific anti-CD248 antibodies after exposure to carrier (Control) or TGFβ for 48 hrs. TGFβ suppresses CD248 in a concentration-dependent manner, with simultaneous increase in phosphorylated Smad2 and ERK1/2. Scale bar = 50 μm.

TGFβ exerts a range of cellular effects by binding to and activating its cognate serine/threonine kinase receptors, TGFβ type I (TGFβRI, ALK-5) and type II (TGFβRII), which in turn mediate intracellular signaling events via canonical Smad-dependent and Smad-independent signaling pathways (e.g. p38 mitogen-activated protein kinase (MAPK) pathway) (for reviews [36-38]). The canonical Smad-dependent pathway results in recruitment and phosphorylation of Smad2 and Smad3 which complex with Smad4 to enter the nucleus and form a transcriptional complex that modulates target gene expression in a context-dependent manner. Diversity in the response to TGFβ signaling is achieved by Smad2/3-independent, “non-canonical” signaling pathways, which may include, among others, activation of combinations of mitogen-activated protein kinases ERK1/2 and p38, PI3K/Akt, cyclo-oxygenase, Ras, RhoA, Abl and Src (for reviews [36-38]). We characterized the pathways by which TGFβ suppresses CD248. MEF were exposed to a range of concentrations of TGFβ (0.1 to 12 ng/ml) for a period of 48 hrs. Western blots of cell lysates showed that TGFβ downregulated the expression of CD248 in a concentration-dependent manner. As expected, TGFβ also induced phosphorylation of Smad2 and Smad3 in a concentration-dependent manner (Figure 2A,B). Confocal microscopy was used to visualize the effects of TGFβ on expression of CD248 by MEF (Figure 2C). At 48 hrs without TGFβ, CD248 was readily detected on the surface of CD248^WT/WT^ MEF, but was entirely absent in TGFβ-treated cells as well as in CD248^KO/KO^ MEF.

**Figure 3:**
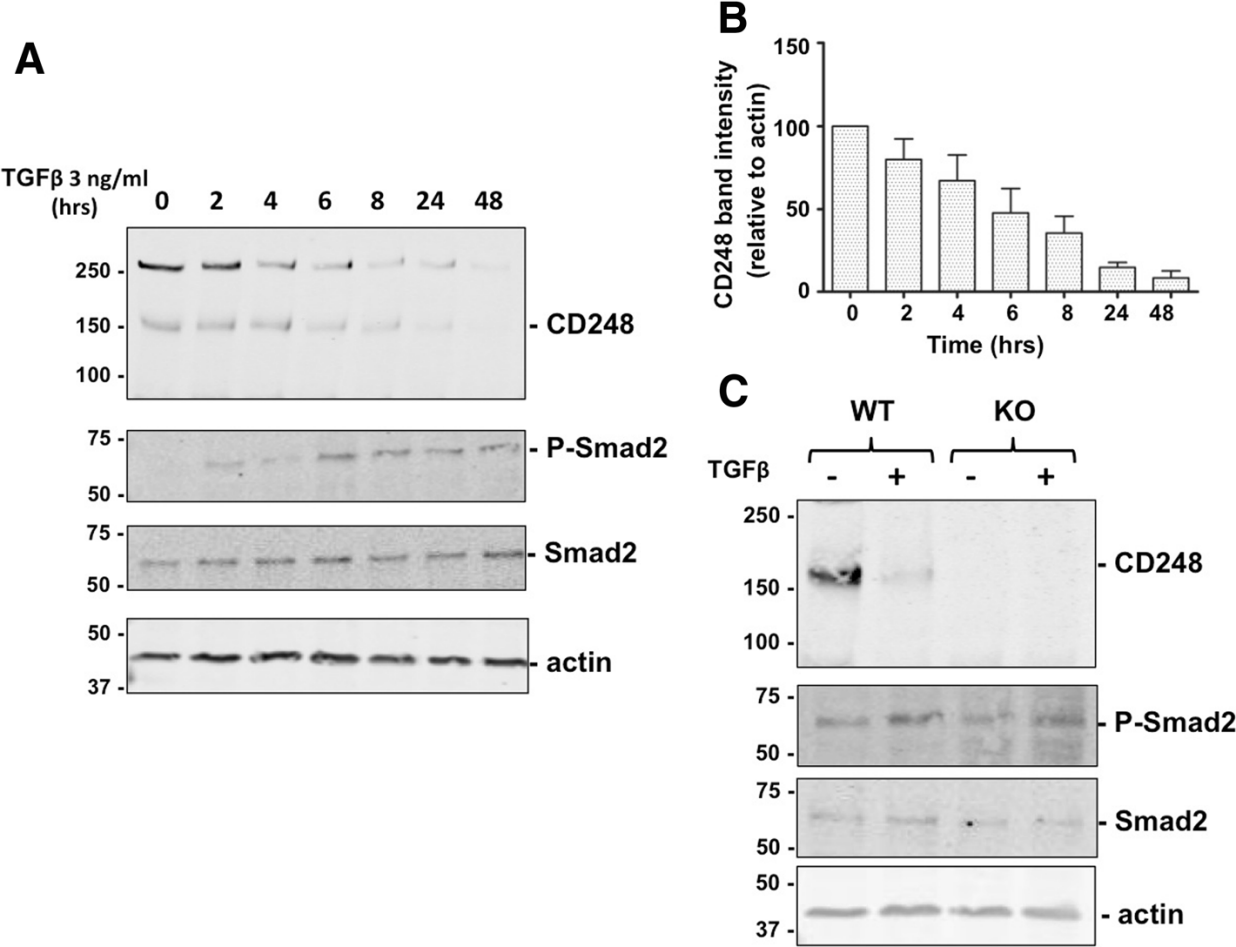
**Temporal response of CD248 to TGFβ. (A)** MEF were incubated for 0-48 hrs with TGFβ 3 ng/ml. Expression of CD248 and phosphorylation of Smad2, were detected by Western blot. **(B)** CD248 expression relative to actin expression was quantified by densitometry (n = 3 experiments) and results were normalized to the no-treatment condition. CD248 expression decreases as Smad2 is phosphorylated. **(C)** CD248^WT/WT^ (WT) or CD248^KO/KO^ (KO) MEF were exposed to TGFβ (0 or 3 ng/ml) for 48 hrs and lysates were Western blotted. Representative blots from 3 experiments are shown. Smad2 and ERK1/2 are phosphorylated in response to TGFβ even in cells that lack CD248.

We next evaluated the temporal response of CD248 to a fixed concentration of TGFβ (3 ng/ml) (Figure 3A,B) and found that CD248 expression was suppressed in a time-dependent manner to <50% by 6 hrs of exposure to TGFβ. Once again, TGFβ induced phosphorylation of Smad2. Notably, as seen in experiments using CD248^KO/KO^ MEF (lacking CD248) (Figure 3C), CD248 was not required for TGFβ-mediated phosphorylation of Smad2, indicating that CD248 is not a co-receptor for TGFβ signaling.

### TGFβ suppresses CD248 mRNA accumulation

**Figure 4:**
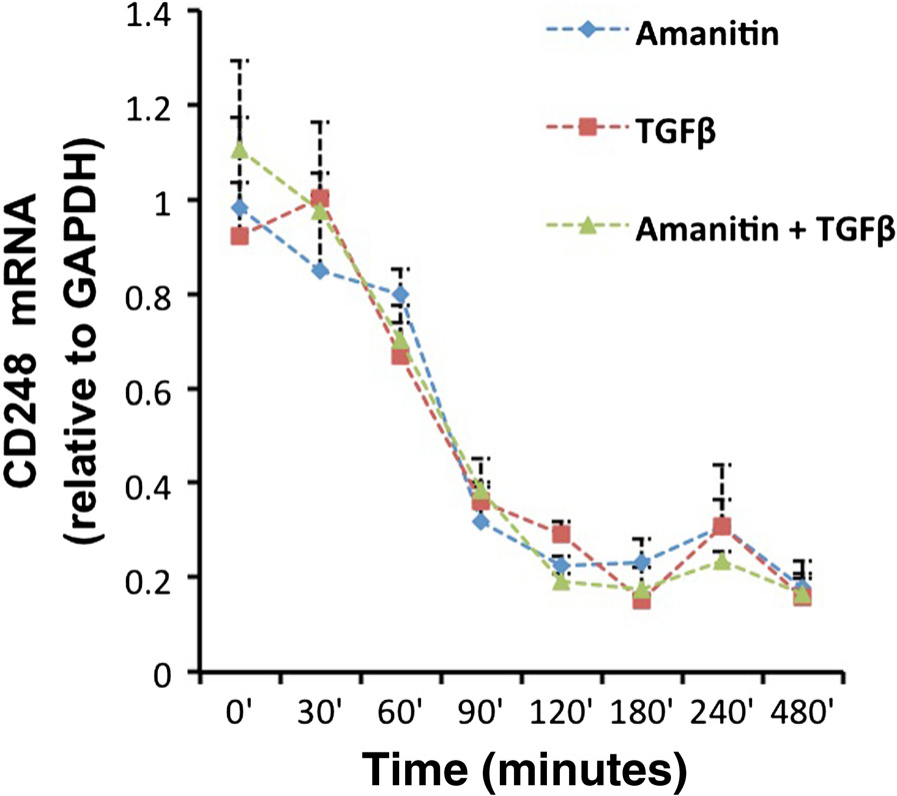
**Stability of CD248 mRNA is unaffected by TGFβ.** MEF were treated with TGFβ 3 ng/ml alone, α-amanatin 20 μg/ml alone, or with a combination of TGFβ and α-amanitin as described in Methods. CD248 mRNA levels, relative to the mRNA levels of the housekeeping gene GAPDH, were quantified at different time intervals by qRT-PCR. Results were normalized from 3 independent experiments, each done in triplicate. The half-life of CD248 mRNA is approximately 75 minutes, which is unaltered by TGFβ.

We evaluated the mechanism by which TGFβ suppresses CD248. CD248 mRNA levels in MEF were quantified by qRT-PCR at different time intervals following exposure of the cells to 3 ng/ml TGFβ. TGFβ suppressed CD248 mRNA levels in a time-dependent manner and by 75 minutes, mRNA accumulation had diminished to ~50% (Figure 4) and was ~20% by 2 hrs.

Using the RNA polymerase II inhibitor, α-amanitin (20 μg/ml), we measured the stability of CD248 mRNA in MEF and assessed whether it is altered by TGFβ. As seen in Figure 4, the time-dependent reduction in CD248 mRNA with α-amanitin alone was almost identical to the pattern seen with TGFβ alone, i.e., the half-life was determined to be approximately 75 minutes. The addition of TGFβ to α-amanitin did not alter the half-life. The findings suggest that TGFβ acts primarily at the level of CD248 transcription and does not alter the stability of CD248 mRNA.

### Suppression of CD248 by TGFβ is mediated by ALK-5 signaling

**Figure 5:**
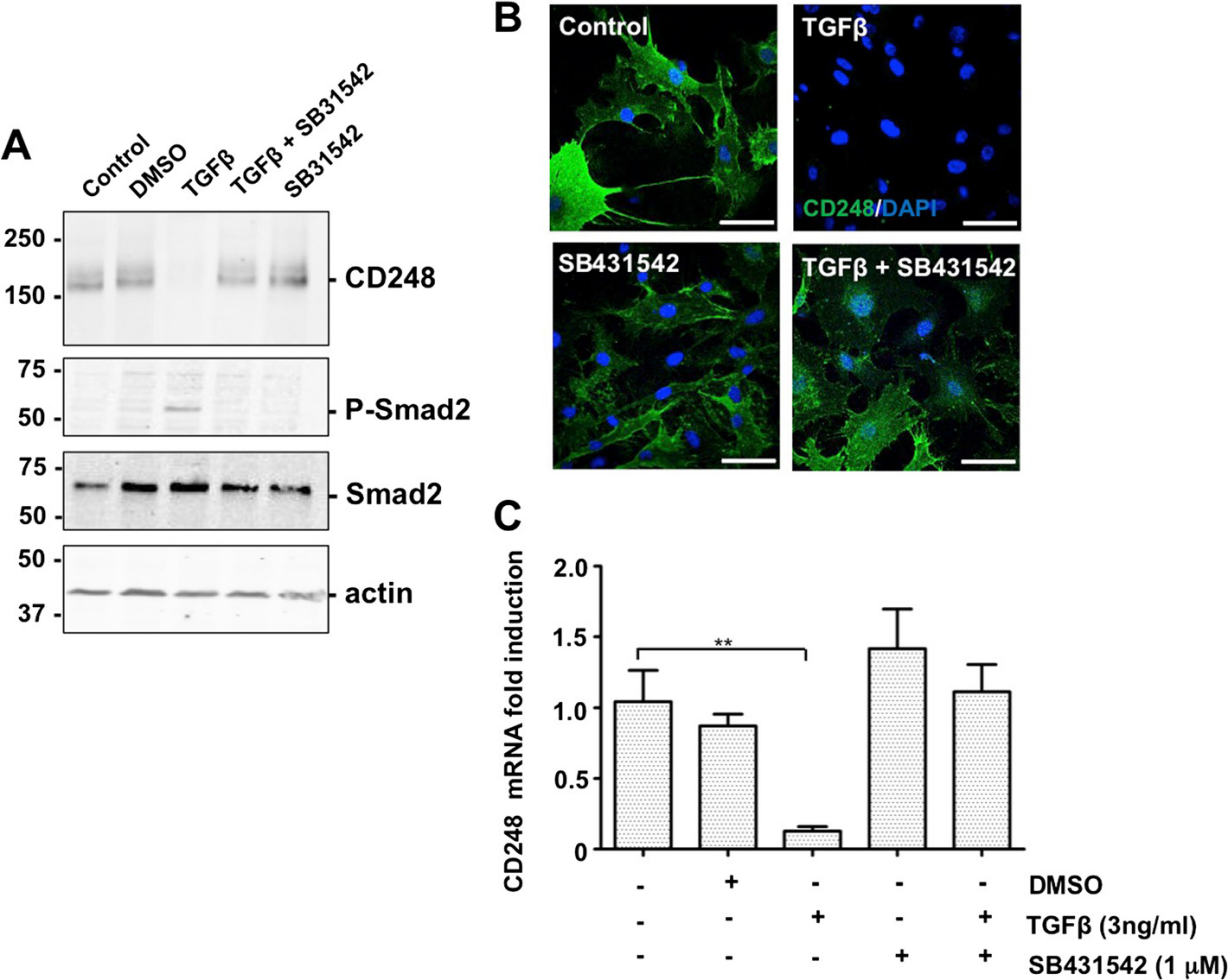
**TGFβ-induced suppression of CD248 is mediated via canonical signaling pathways. (A, B, C)** MEF were incubated for 48 hrs with TGFβ 3 ng/ml and the ALK-inhibitor SB431542 1 μM either singly or in combination. Controls included carriers for SB431542 (DMSO) or for TGFβ (0.1% BSA). **(A)** Western blots and **(B)** immunofluorescence were used to detect expression of CD248 (green). **(C)** CD248 mRNA levels were also quantified (n = 3 experiments, each in triplicate; *p < 0.05). Results indicate that TGFβ-mediated suppression of CD248 protein and mRNA requires integrity of canonical ALK5-Smad2 signaling pathway. Scale bar = 50 μm.

In MEF, TGFβ reportedly signals exclusively through complexes involving ALK5 [39]. SB431542 is a selective inhibitor of TGFβ superfamily type I activin receptor-like kinase (ALK) receptors, ALK4, ALK5 and ALK7, which does not affect components of the ERK, JNK, or p38 MAP kinase pathways [40]. We tested whether ALK5 is required for TGFβ-mediated suppression of CD248. MEF were incubated with the inhibitor (1 μM) for 1 hr prior to the addition of 3 ng/ml TGFβ. Expression of CD248 at 48 hrs was assessed by Western blot, immunofluorescence analysis and qRT-PCR (Figure 5A-C). When added alone, neither the inhibitor SB431542 nor its vehicle DMSO, had any effect on CD248 expression. As before, TGFβ dramatically suppressed CD248, while simultaneously inducing phosphorylation of Smad2 (Figure 5A). This effect of TGFβ was entirely abrogated by preincubation of the cells with SB431542. Thus, addition of TGFβ down-regulates CD248 via activation of ALK-5.

### TGFβ-mediated suppression of CD248 is independent of ERK1/2 and p38 signaling

**Figure 6:**
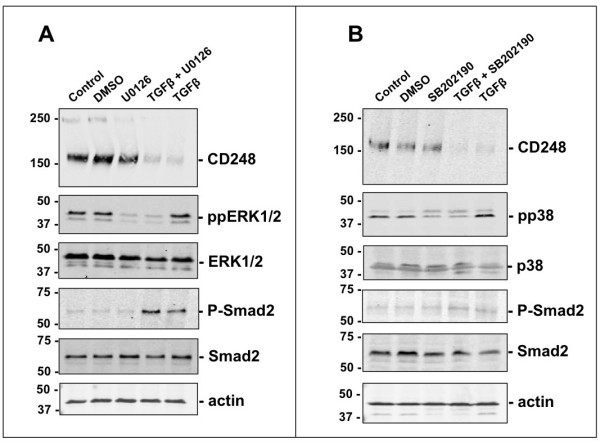
**TGFβ-mediated suppression of CD248 via ALK5 is specific. (A, B)** MEF were incubated with TGFβ (3 ng/ml) for 48 hrs in the presence or absence of the inhibitor of phosphorylated ERK1/2, U0126 10 μM **(A)** or phosphorylated p38, SB202190 10 μM **(B)**. Representative Western blots from 3 independent experiments are shown and were used to assess the effect on CD248 expression. TGFβ-coupling to either ERK1/2 or to p38 is not involved in its suppressive effects on CD248.

We also tested whether suppression of CD248 expression by TGFβ is mediated via one or more non-canonical Smad2/3-independent pathways. Using U0126, a specific inhibitor of ERK1/2 phosphorylation [41], we showed that TGFβ does not rely on signaling via ERK1/2 to suppress CD248 (Figure 6A). In a similar manner, using the p38 inhibitor, SB202190 [42], we also demonstrated that phosphorylation of p38 is not required for TGFβ to downregulate expression of CD248 (Figure 6B). Thus, in MEF, TGFβ suppresses CD248 expression via signaling pathways that do not require activation of these two Smad2/3-independent pathways.

### Regulation of CD248 by Bone morphogenic protein 2 (BMP2) and Activin

**Figure 7:**
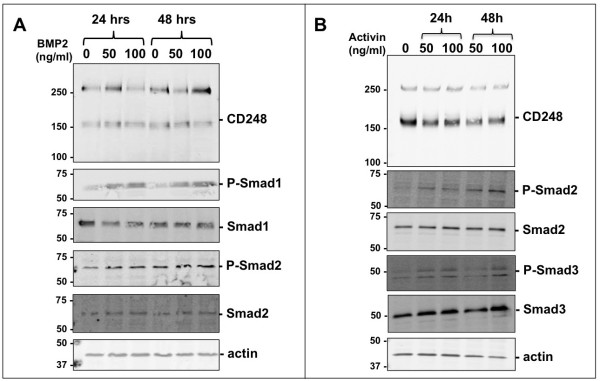
**Regulation of CD248 by BMP-2 and Activin.** MEF were incubated with different concentrations of BMP2 **(A)** or activin **(B)** for 24 or 48 hrs. Representative Western blots from 3 independent experiments are shown and were used to assess the effect on CD248 expression.

The TGFβ family of cytokines comprises over 35 members, including the prototypic TGFβ isoforms (TGFβ1, β2, β3), bone morphogenic proteins (BMPs), growth and differentiation factors, activins and nodal. These regulate cell survival, proliferation, differentiation, adhesion, migration and death in a cell type-and context-dependent manner. To further assess the specificity of action of TGFβ on CD248 expression, we tested whether BMP2 and activin had similar effects. MEF were treated for 24 and 48 hrs with 50 and 100 ng/ml of activin or BMP2 (Figure 7A). At these concentrations of BMP2, Smad1 was, as expected, phosphorylated, while Smad2 was not [43]. Notably, BMP2 had no effect on CD248 expression, and thus does not participate in its regulation under these conditions. Activin induced phosphorylation of Smad2, which reportedly occurs via ALK-4/7 activation [44] (Figure 7B). In contrast to TGFβ, activin caused only a slight reduction in CD248 expression after 48 hrs of exposure.

### Cancer cell lines are resistant to TGFβ suppression of CD248

**Figure 8:**
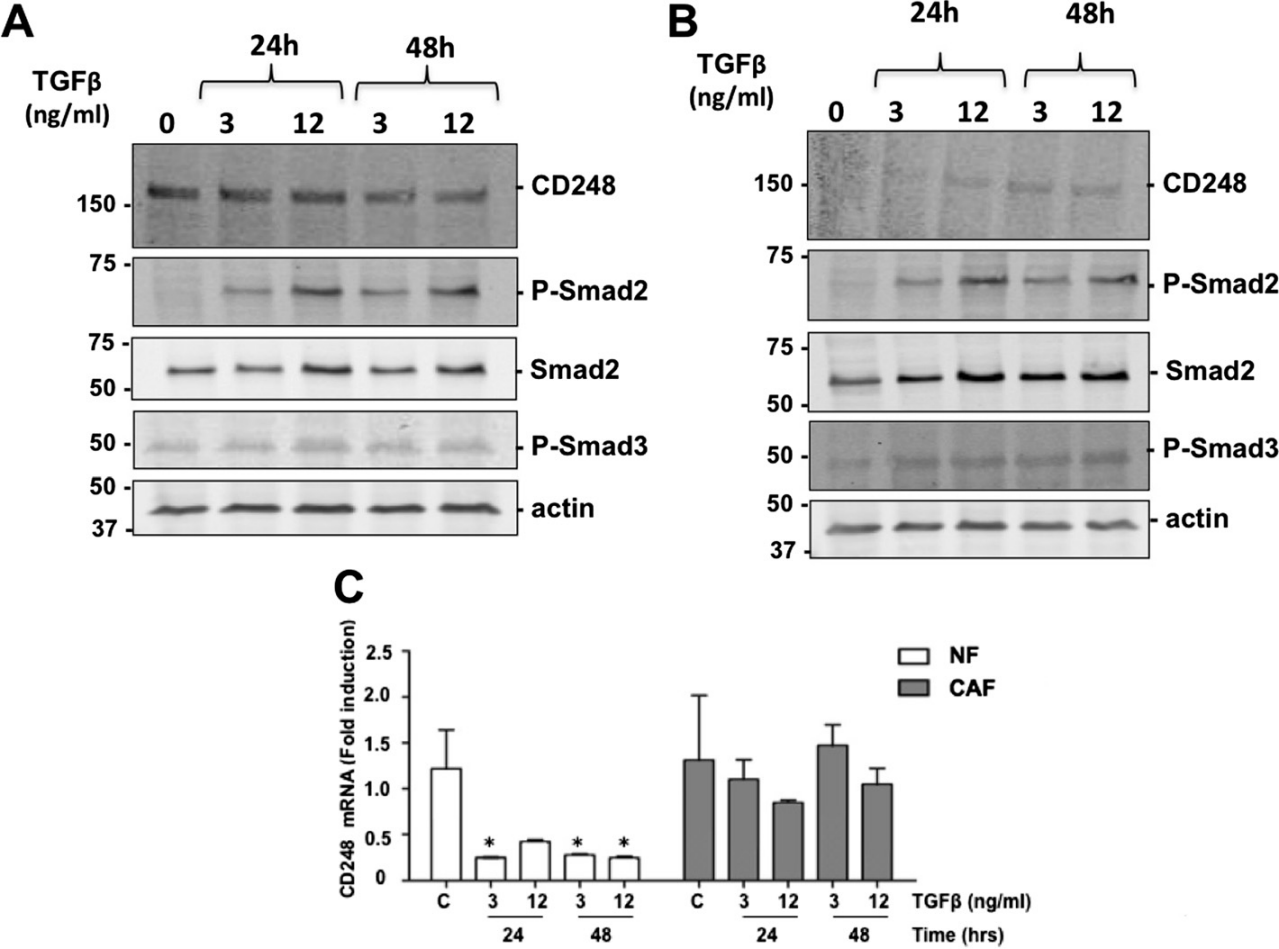
**Regulation of CD248 in cancer cells. (A, B)** Wehi-231 **(A)** and A20 **(B)** mouse lymphoma cells were incubated with different concentrations of TGFβ for 24 or 48 hrs and lysates were assessed by Western immunoblot. CD248 levels were minimally affected in spite of phosphorylation of Smad2. Results are representative of 3 independent experiments. **(C)** Normal fibroblasts (NF) and cancer associated fibroblasts (CAF) from murine mammary tissue were exposed to TGFβ for 24 or 48 hrs and CD248 mRNA levels were quantified and normalized to levels from untreated NF. CD248 mRNA levels in NF were significantly suppressed by TGFβ, whereas there was no effect on CD248 in CAF. *p < 0.05, n = 3.

Since elevated CD248 is associated with tumorigenesis, we tested whether TGFβ could suppress CD248 in tumor cell lines as effectively as in the healthy non-cancerous cells examined above. Mouse B lymphoma cell lines, Wehi-231 and A20 were incubated with TGFβ at concentrations of 3 ng/ml and 12 ng/ml for 24 hrs and 48 hrs (Figure 8). Under these conditions, SMAD2 was phosphorylated, with minimal effect on Smad3 phosphorylation. In both the Wehi-231 cells (Figure 8A) and the A20 cells (Figure 8B), there was no significant suppression of CD248 expression in response to TGFβ. Indeed, in the latter, there was a slight increase in CD248 in response to the TGFβ.

We also examined the effect of TGFβ on the expression of CD248 by normal and cancer associated fibroblasts (NF and CAF, respectively) that were derived from mouse mammary tissues [33]. Protein levels of CD248 were relatively low in both of these cell lines, making it difficult to assess changes by Western blot. CD248 mRNA levels were therefore quantified by qRT-PCR (Figure 8C). Following exposure of the cells to 3 ng/ml or 12 ng/ml TGFβ for 24 and 48 hrs, CD248 mRNA accumulation was significantly suppressed in the NF, while in contrast, there was no effect on CD248 mRNA levels in the CAF. Overall, the preceding findings indicate that the expression of CD248 in cancer cells is resistant to regulation by TGFβ.

## Discussion

Since the discovery of CD248 [45], clinical and genetic evidence has pointed to it as a promoter of tumor growth and inflammation (reviewed in [2]). Increased expression of CD248 is detected in stromal cells surrounding most tumors, and high levels often correlate with a poor prognosis [20,23]. Means of interfering with the tumorigenic effects of CD248 have eluded investigators due to a lack of knowledge surrounding the regulation of CD248. This has limited opportunities for the design of innovative therapeutic approaches. In this report, we show that expression of CD248 by non-cancerous cells of mesenchymal origin is specifically and dramatically downregulated at a transcriptional and protein level by the pleiotropic cytokine, TGFβ, and that the response is dependent on canonical Smad2/3-dependent signaling. Notably, CD248 expression by cancer cells and cancer associated fibroblasts is not altered by TGFβ. The findings suggest that a TGFβ-based strategy to suppress CD248 may be useful as a therapeutic intervention to prevent early stage, but not later stage, tumorigenesis.

Members of the TGFβ family regulate a wide range of cellular processes (e.g. cell proliferation, differentiation, migration, apoptosis) that are highly context-dependent, i.e., stage of development, stage of disease, cell/tissue type and location, microenvironmental factors, and epigenetic factors. Under normal conditions, TGFβ plays a dominant role as a tumor suppressor at early stages of tumorigenesis, inhibiting cell proliferation and cell migration (reviewed in [46,47]). TGFβ ligands signal via TGFβRI (ALK-5) and TGFβRII. A third accessory type III receptor (TGFβRIII) lacks kinase activity, but facilitates the tumor-suppressor activities of TGFβ. TGFβ binds to TGFβRII which transphosphorylates ALK-5. In canonical signaling, ALK-5 then phosphorylates Smad2 and Smad3, inducing the formation of heteromeric complexes with Smad4, for translocation into the nucleus, interaction with transcription factors, and regulation of promoters of several target genes [48,49]. Disruption of TGFβ signaling has been associated with several cancers and a poor prognosis [47], and mice that lack TGFβ spontaneously develop tumors and inflammation [50].

TGFβ signaling is not, however, restricted to Smads 2 and 3, but can couple to non-canonical (Smad2/3-independent) effectors [48,51-54]. Recent data support the notion that canonical signaling favours tumor suppression, while non-canonical signaling tips the balance, such that TGFβ switches to become a promoter of tumor growth, invasion and metastasis, overriding the tumor-suppressing activities transmitted via Smad2/3. This dichotomous nature is known as the “TGFβ Paradox”, a term coined to describe the conversion in function of TGFβ from tumor suppressor to tumor promoter [55-57]. The mechanisms underlying this switch are steadily being delineated, as regulation of the multiple effector molecules that are coupled to TGFβ are identified and characterized (reviewed in [47]). Our findings suggest that CD248 may be one such TGFβ-effector molecule that undergoes a context-dependent change in coupling, and thus may be a potential therapeutic target.

Upon determining that TGFβ suppresses CD248, we first showed that the response is dependent on Smad 2 signaling. This is consistent with the almost undetectable levels of CD248 in normal tissues, its expression presumably held in check at least in part by TGFβ’s tumor suppressor properties. The fact that TGFβ induces phosphorylation of Smad2 in MEF that lack CD248, indicates that CD248 is not required for Smad2 phosphorylation. Rather, in the TGFβ-signaling pathway, CD248 is positioned “downstream” of Smad2/3 phosphorylation. We also showed that CD248 is downregulated by TGFβ primarily at a transcriptional level, and without affecting the stability of its mRNA. We have not determined which regions of the CD248 promoter are required for TGFβ-induced suppression. However, intriguingly, the murine promoter of the CD248 gene contains the sequence 5′-TTTGGCGG (position -543 to -536) [5] that overlaps with a consensus E2F transcription factor binding site. This is almost identical to the unique Smad3 DNA binding site in the *c-myc* promoter that is crucial for TGFβ-induced gene suppression [58]. Detailed mapping of the promoter will provide insights into precisely how CD248 is regulated by TGFβ.

We also examined whether TGFβ coupling to non-canonical effector molecules, ERK1/2 and p38, alters expression of CD248. Neither ERK1/2 nor p38, pathways implicated in TGFβ-induced metastasis, affected CD248 expression. Thus, based on current data, TGFβ-induced suppression of CD248 occurs primarily, if not exclusively, via canonical Smad2/3 signaling.

The specificity of the response of CD248 to TGFβ extends beyond Smad2/3-related signaling. In a survey of growth factors and cytokines, we could not identify other factors that similarly suppress (or conversely, increase) CD248 expression in MEF, 10 T1/2 cells or primary vascular smooth muscle cells. Even BMP2 and activin, members of the TGFβ superfamily and pleiotropic cytokines that also exhibit tumor promoter and suppressor activities, had little effect on CD248 expression. Although our survey was limited in range, concentration and time of exposure, the findings suggest specificity, and highlight the central role that TGFβ likely plays in regulating expression of CD248 in non-cancerous cells.

Most notably, in two tumor cell lines and in cancer associated fibroblasts, the regulation of expression of CD248 was resistant to TGFβ. Indeed, in these cells, TGFβ neither decreased nor increased CD248, suggesting a decoupling of the regulatory link between TGFβ and CD248. Thus, with the switch from a tumor suppressor to a tumor promoter, TGFβ loses it ability to regulate CD248. Although TGFβ does not appear to directly participate in enhancing CD248 expression during late tumorigenesis, loss of its ability to suppress CD248 may be relevant in tumor progression and metastasis.

## Conclusions

We have shown that the tumor suppressor properties of TGFβ, observed in early stage cancer, are likely mediated in part via suppression of CD248, the latter which is mediated via canonical Smad-dependent pathways. Upregulation of CD248 might be an early detection marker of tumor growth and metastasis, and may be valuable in monitoring TGFβ-based therapies. The clinical relevance of understanding how CD248 is regulated is highlighted by ongoing Phase 1 and 2 clinical trials in which the anti-CD248 antibody, MORAb-004, is being tested for efficacy in solid tumors and lymphomas (http://www.clinicaltrials.gov). Delineating the molecular mechanism(s) by which TGFβ loses its ability to suppress CD248 will be key for the design of additional therapeutic interventions to prevent and/or reduce CD248-dependent tumor cell proliferation and metastasis.

## Competing interests

The authors declare that they have no competing interests.

## Authors’ contributions

SSB helped design and perform the experiments and wrote the manuscript. YV helped design the studies and prepare the manuscript. AX, AO and VL provided technical support. FC prepared and provided normal and cancer associated fibroblasts. EMC supervised, directed and designed all studies and wrote the manuscript. All authors read and approved the final manuscript.

## Pre-publication history

The pre-publication history for this paper can be accessed here:

http://www.biomedcentral.com/1471-2407/14/113/prepub
